# Méningo-encéphalocèle sphénoïdal chez un adulte

**DOI:** 10.11604/pamj.2020.37.119.25543

**Published:** 2020-10-05

**Authors:** Mehdi Hasnaoui, Rihab Guizani

**Affiliations:** 1 Department of Otolaryngology-Head and Neck Surgery, Tahar Sfar Hospital, Mahdia, Tunisia

**Keywords:** Méningocèle, encéphalocèle, sinus sphénoïdal, tomodensitométrie, imagerie par résonance magnétique, Meningocele, encephalocele, sphenoid sinus, tomodensitometry, magnetic resonance imaging

## Abstract

The incidence of sphenoid sinus meningoencephalocele is very low. It is estimated one person out of 700 000 live births. This clinical entity is rarer in adults. We here report the case of a 51-year old woman presenting with feeling of intranasal heaviness that had progressed since the young age without rhinorrhea or nasal obstruction. She had no dysosmia or headache. She did not have a history of recurrent meningitis, skull base surgery or head trauma. Both nasal fossae were free. Computed tomography (CT) scan of the facial skeleton showed subtotal filling of the right sphenoid sinus, with spontaneously hyperdense content at places, without significant contrast enhancement associated with lysis of the lateral wall of the right sphenoid sinus. Brain and facial skeleton magnetic resonance imaging (MRI) showed dehiscence of the lateral wall of the right sphenoid sinus with herniation of the cerebrospinal fluid presenting hyposinal on T1, hypersignal on T2, cerebral parenchyma in T1 and T2 isosignal. We opted for therapeutic abstention because the patient was asymptomatic.

## Image en médecine

L´incidence du méningo-encéphalocèle sphénoïdal est très faible. Elle est estimée à une personne sur 700 000 naissances vivantes. Cette entité clinique est plus rare chez l'adulte. Notre patiente est âgée de 51 ans. Elle a consulté pour une sensation de pesanteur intra-nasal évoluant depuis le jeune âge sans rhinorrhée ni obstruction nasale. Elle n´avait pas de dysosmie ni de céphalée. Elle n´avait pas des antécédents de méningite à répétition ni de chirurgie de la base du crâne ni de traumatisme crânien. Les deux fosses nasales étaient libres. Le scanner du massif facial a montré un comblement subtotal du sinus sphénoïdal droit, à contenu spontanément hyperdense par endroit, sans prise de contraste significative associé à une lyse de la paroi latérale de sinus sphénoïdal droit. L´IRM cérébrale et du massif facial a montré une déhiscence de la paroi latérale du sinus sphénoïdal droit avec en regard une hernie du liquide céphalo-rachidien (LCR) en hyposignal T1, hypersignal T2 et du parenchyme cérébral en isosignal T1 et T2. La conduite à tenir était l´abstention thérapeutique vu que la patiente était asymptomatique.

**Figure 1:**
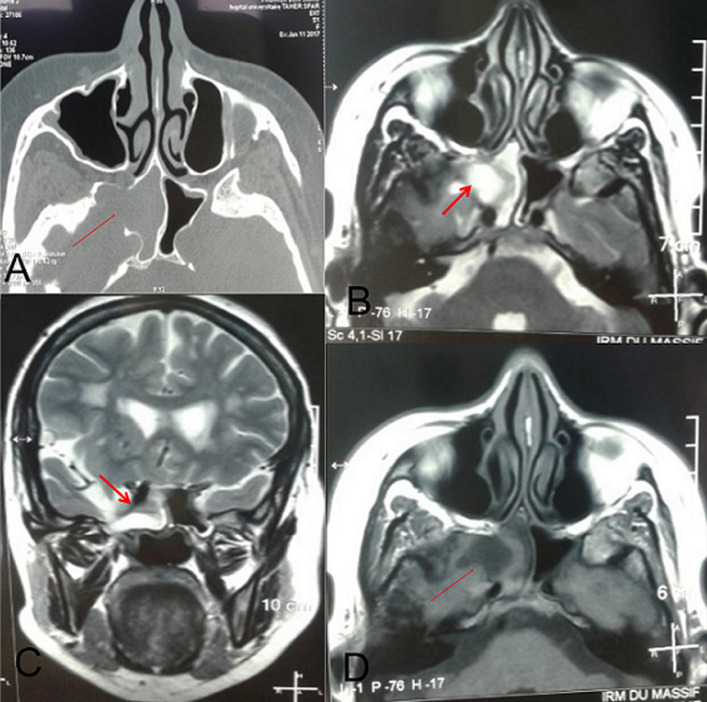
A) scanner du massif facial en coupe axiale montrant un comblement du sinus sphénoïdal droit associé à une lyse de la paroi latérale de sinus sphénoïdal droit, IRM cérébrale et faciale en coupe axiale T1; B) coupe coronale T2; C) déhiscence de la paroi latérale du sinus sphénoïdal droit avec en regard une hernie du LCR en hyposignal T1, hypersignal T2 et du parenchyme cérébral en isosignal T1 et T2; D) T2

